# Identification, Design and Bio-Evaluation of Novel Hsp90 Inhibitors by Ligand-Based Virtual Screening

**DOI:** 10.1371/journal.pone.0059315

**Published:** 2013-04-02

**Authors:** JianMin Jia, XiaoLi Xu, Fang Liu, XiaoKe Guo, MingYe Zhang, MengChen Lu, LiLi Xu, JinLian Wei, Jia Zhu, ShengLie Zhang, ShengMiao Zhang, HaoPeng Sun, QiDong You

**Affiliations:** 1 State Key Laboratory of Natural Medicines, China Pharmaceutical University, Nanjing, China; 2 Jiang Su Key Laboratory of Drug Design and Optimization, China Pharmaceutical University, Nanjing, China; 3 Department of Medicinal Chemistry, China Pharmaceutical University, Nanjing, China; Wake Forest University, United States of America

## Abstract

Heat shock protein 90 (Hsp90), whose inhibitors have shown promising activity in clinical trials, is an attractive anticancer target. In this work, we first explored the significant pharmacophore features needed for Hsp90 inhibitors by generating a 3D-QSAR pharmacophore model. It was then used to virtually screen the SPECS databases, identifying 17 hits. Compound **S1** and **S13** exhibited the most potent inhibitory activity against Hsp90, with IC_50_ value 1.61±0.28 μM and 2.83±0.67 μM, respectively. Binding patterns analysis of the two compounds with Hsp90 revealed reasonable interaction modes. Further evaluation showed that the compounds exhibited good anti-proliferative effects against a series of cancer cell lines with high expression level of Hsp90. Meanwhile, **S13** induced cell apoptosis in a dose-dependent manner in different cell lines. Based on the consideration of binding affinities, physicochemical properties and toxicities, 24 derivatives of **S13** were designed, leading to the more promising compound **S40**, which deserves further optimization.

## Introduction

Heat shock protein 90 (Hsp90) is a member of chaperone protein family, which play a crucial role in regulating numerous cellular processes, including protein folding, cell apoptosis, and stress resistance [1]–[2]. As an ATPase-dependent protein folding molecular chaperone, Hsp90 functions with a cluster of co-chaperones to facilitate the stability and biological function of numerous client proteins, many of which are related to carcinogenesis, such as Met, Erb-B2, VEGF, Akt, EGFR and Bcr-Abl [3]–[6]. Several Hsp90 clients are notorious oncogenes (Raf-1, Akt, cdk4, Src, Flt-3, hTert, c-Met, etc.), and five of them are clinically validated cancer targets: HER-2/neu, Bcr-Abl, estrogen receptor, androgen receptor, and VEGFR [7]–[10]. Such a major advantage of Hsp90 inhibitors is that they simultaneously attack several pathways which are necessary for cancer development, reducing the likelihood of the tumor acquiring resistance [11]. Additionally, Hsp90 inhibitors have shown selectivity for cancer cells [12]–[13].This can be explained for several reasons: (1) the active Hsp90 in cancer cells has higher affinity to Hsp90 inhibitors than the latent form in normal cells, leading to an increased accumulation of inhibitor in cancer cells; (2) Hsp90 is overexpressed in many types of cancers in humans [12]–[13]. For these reasons, Hsp90 has emerged as a promising target for anti-cancer drug development.

The role of Hsp90 in oncogenic transformation has not been appreciated until the discovery of pharmacological agents that selectively inhibit its function [14]–[16]. The therapeutic potential of Hsp90 inhibitors has been verified by the initial success of the natural product 17-allylamino-17-demethoxygeldanamycin (**17-AAG**) in several Phase I and Phase II clinical trials in cancers therapy [17]–[21]. Additionally, other synthetic Hsp90 inhibitors such as purine derivative **BIIB021** and isoxazole derivative **VER-52296/NVP-AUY922** also have entered clinical trials [22]–[23]. Although it has been under clinical trials for many years, 17-AAG encounters a lot of severe problems including poor solubility, liver toxicity and multidrug resistance (MDR) caused by pglycoprotein (P-gp) efflux pump [15], [24]. These issues highlight a critical need for novel and improved inhibitors to overcome the limitations.

Computer-assisted techniques, such as pharmacophore-based or docking-based virtual screening has emerged as an effective tool for novel active compound identification. Meanwhile, the crucial information in target-ligand interaction revealed by these methods also has improved the reasonability and accuracy of molecular design. A large number of successful applications in medicinal chemistry have demonstrated the importance of these methods in drug design [25]–[27].

With the aim of acquiring novel scaffolds of Hsp90 inhibitors, in the present study, a 3D pharmacophore model, Hypo1, was generated on the basis of 18 known Hsp90 inhibitors. The model was validated by external dataset containing 30 known Hsp90 inhibitors and then used for virtual screening. Hit compounds from SPECS database were validated by molecular docking and 17 retained compounds were bought and subjected to biological evaluation. Compound **S1** and **S13** with novel scaffolds exhibited potent Hsp90 inhibitory activity, with IC_50_ 1.61±0.28 μM and 2.83±0.67 μM, respectively. The two compounds also showed good cytotoxicity against a series of cancer cell lines. **S13**-induced cell morphological change of MCF-7 cancer cells was observed. A panel of the client proteins, including Her2, Src, Akt, ERK, c-Raf and Hif-1α, were also found to be downregulated by **S13**. Using **S13** as lead, 24 novel derivatives were designed and evaluated based on their binding affinities, physicochemical properties and toxicities, leading to a more promising compound **S40**, which deserves further optimization.

## Materials and Methods

### General methodology and materials

The following program were used in the manuscript: Discovery Studio 3.0 software package for pharmacophore model generation (DS, Accelrys Inc., San Diego, USA); Gold 5.0 program for molecular docking (CCDC, UK); Derek 2.0.3 for the toxicities prediction (Lhasa Inc., UK); MarvinSketch 5.10.0 for the physicochemical properties prediction (Chemaxon Ltd., USA). All the calculation and display of the molecules were performed on Dawning 560I workstation.

The following materials were used for the biological evaluation: The PET-28a HSP90 expression vector was constructed. The Ni^2+^-nitrilo-triacetic acid (NTA) agarose was purchased from General Electric (USA). 17-Dimethylamino-ethylamino-17-demethoxygeldanamycin (17-DMAG) and **AT13387** were from Selleck (USA). Isopropyl-1-thio-d-galactopyranoside (IPTG) was purchased from Sigma (St. Lousi. MO). Antibodies of β-Actin, Hsp70, Hsp90, c-Raf, Akt, Src, Phospho-Src, ERK and Her-2 were purchased from Cell Signaling Technology (Danvers, MA). Anti-p-Akt(S473) was purchased from Signalway Antibody (Baltimore, MD). HIF-1α antibody was purchased from R&D systems. The stock solution of Hsp90 was prepared in a 20 mM Tris-Cl buffer with a pH 7.4, and concentration was fixed at 5.0 μM. Hsp70 (human, recombinant, ALX-201-214-C025) with ATPase activity is supplied by Enzo Life science (Waterloo, Australia). The screened out compounds were purchased from SPECS and dissolved in DMSO (Sigma, St. Louis, MO) to make a final concentration of 10^−2^ mol/L and were stored at −20°C. The tumor cells HCT116, HepG2, MDA-MB-231, MCF-7, SKBr3 and A549 were purchased from Cell Bank of Shanghai Institute of Biochemistry and Cell Biology, Chinese Academy of Science. The water used in the experiments was thrice-distilled and all other materials were of analytical reagent grade.

### Training set and test set selection

In the course of construction of pharmacophore model, the selection of training set will deeply affect the accuracy and reliability of the model. According to literature reported rules [28], the principles for selecting training set are as follows: a) the number of compound in the training set is more than sixteen; b) all the biological data were obtained using similar method, and the data can cover an activity range of at least four orders of magnitude (at least 4 compounds for each order of magnitude); c) the set must contain structurally diverse compounds to ensure that each compound can provide new features to the pharmacophore model.

**Figure 1:**
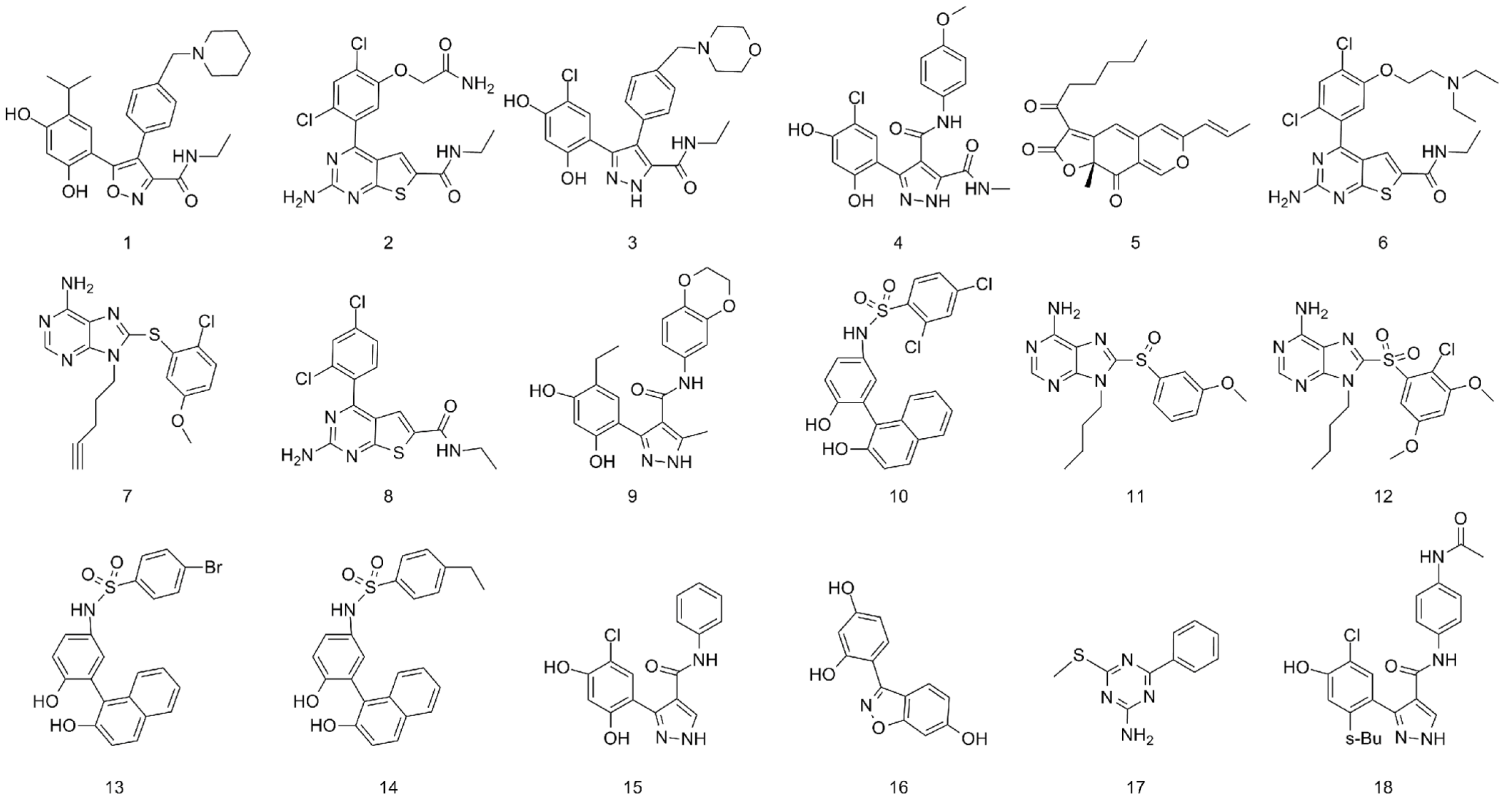
2D Chemical structures of 18 training set used to obtain HypoGen pharmacophore hypotheses.

**Figure 2:**
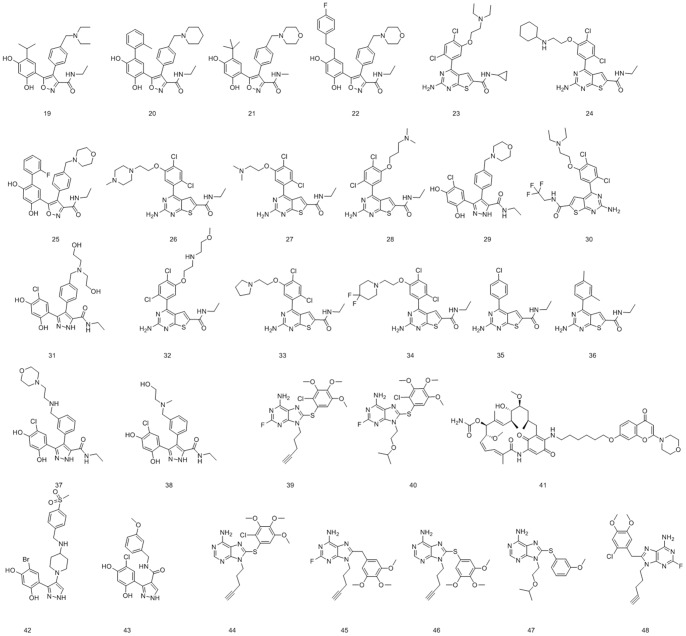
2D chemical structures of 30 test set molecules for the pharmacophore model validation.

In this study, the activity data for training set and test set molecules were obtained from several literatures [23], [29], [30]. According to the above rules, eighteen compounds (No. **1–18** in Figure 1), with IC_50_ ranging from 0.006 μM to 31.5 μM, were used as training set to generate HypoGen hypotheses. Another thirty Hsp90 inhibitors with diverse activities and structures were selected as test set to validate the pharmacophore model (No. **19–48** in Figure 2). The biological data (represented as IC_50_ values) of these inhibitors were determined under a similar experimental condition by using a fluorescence polarization (FP) competitive binding assay method [23], [29], [30]. All molecules were built using Prepare Ligands module and Minimize Ligands module in DS. Multiple conformations of each compound were generated by using Diverse Conformation Generation protocol with an energy threshold of 20 kcal/mol and a maximum of 255 conformers.

### Generation of pharmacophore hypotheses with 3D-QSAR pharmacophore generation (HypoGen)

HypoGen attempts to develop SAR hypothesis models from a set of molecules for which activities (IC_50_, K_i_, etc.) on a given biological target have been measured. Based on the chemical features of compounds in the training set, we selected the following chemical functions in the feature dictionary: hydrogen bond acceptor (HBA), hydrogen bond donor (HBD), hydrophobic (HY), and hydrophobic aromatic (HYAr) groups. A default uncertainty factor of 3 for each compound was then defined for the representation of the ratio range of uncertainty in the activity value based the expected statistical straggling of biological data collection. Pharmacophore models were then generated by using 3D-QSAR Pharmacophore Generation protocol, and the top ten unique pharmacophore models were finally exported.

### Validation of Pharmacophore Model

The quality of 3D-QSAR pharmacophore models can be best described in terms of two cost-related parameters provided by Catalyst program. The first one is the cost of an ideal hypothesis, which is a lower bound on the cost of the simplest possible hypothesis that still fits the data perfectly; the second one is the cost of the null hypothesis, which presumes that statistically significant structure was not included in the data. For a reliable pharmacophore model, the total cost should be close to the fixed cost, and there should be a significant difference between null and total cost. Further, a value of 40–60 bits for the unit of cost difference implies a 75–90% probability of the correlation between experimental and predicted activities [28].

To verify if the hypothesis can also predict the activity of external compounds, a test set consisting of other 30 molecules (No. **19–48** in Figure 2) in different activity and structural classes were applied to examine the established hypothesis by using the same way as in the training set. All test set molecules were built and minimized as the training set molecules. For further evaluation of the quality of the generated Pharmacophore model, we selected the crystal structure of Hsp90-ligand complex (id: 2vcj) from the Protein Data Bank (PDB). The crystal conformation of the ligand were extracted from the complex and then overlapped onto the pharmacophore model by using the Ligand Pharmacophore Mapping protocol implemented in the Discovery Studio package.

### Database searching

Virtual screening of commercial available databases forms one aspect of an efficiently approach to find novel and potential leads for further development [31]. In this study, the best-ranked four-featured pharmacophore model, Hypo1, was used to screen SPECS database using 3D Database Search protocol in DS. All database searching was performed by using the Best/Flexible search option. The compounds mapped all the critical features in Hypo1 were retained as hit. Hit compounds with the fit values over 7.0 were analyzed for their drug-likeness properties by using Lipinski rule of 5 and ADMET (Absorption, Distribution, Metabolism, Excretion and Toxicity) filters in DS. Compounds those passed all of the screening experiments were retained for molecular docking.

### Binding pattern prediction

Docking studies were carried out using GOLD docking software 5.0 [32], which use a powerful genetic algorithm (GA) method for conformation search and docking. It is widely regarded as one of the best docking programs. In the present study, the Hsp90-ligand complex (PDB id: 3d0b) were selected for docking studies. Residues around the original ligand (radius 8.0Å) were defined as the active site, which completely covered the ATP binding pocket of Hsp90. Before docking different possible stereoisomers, ionized forms and conformations of ligands were prepared by Prepare Ligands protocol in DS at pH 7.0±0.2. Docking studies were performed using the standard default settings with 10 GA runs on each molecule. For each of the GA runs, a maximum of 125000 operations were performed. With respect to ligand flexibility special care has been taken by including options such as flipping of ring corners, amides, pyramidal nitrogens, secondary and tertiary amines, and rotation of carboxylate groups, as well as torsion angle distribution and postprocess rotatable bonds as default. The annealing parameters were used as default cutoff values of 3.0 Å for hydrogen bonds and 4.0 Å for van der Waals interactions. Hydrophobic fitting points were calculated to facilitate the correct starting orientation of the compound for docking by placing the hydrophobic atoms appropriately in the corresponding areas of the active site. When the top three solutions attained root-mean-square deviation (rmsd) values within 1.5 Å, docking was terminated. Chem-Score, a scoring function of the software, is a dimensionless fitness value that takes into account the intra- and intermolecular hydrogen bonding interaction energy, van der Waals energy, and ligand torsion energy. Finally, 17 compounds were retained and purchased from SPECS database with purity >95% (LCMS).

### Physicochemical properties and toxcities prediction of the designed compounds

The physicochemical properties including CLogP, polar surface area (PSA) of the compounds were calculated by Chemaxon MarvinSketch 5.10.0. Weighted method was used and all the parameters were set as default. The toxicities of the compounds were predicted by using Derek 2.0.3. All the endpoints in mammal were selected for prediction and the data were saved as PDF format.

### Anti-proliferation activity

Cell viabilities were measured by a colorimetric assay using 3-(4, 5-dimethylthiaz-ol-2-yl)-2,5-diphenyltetrazoliumbromide (MTT, *Sigma, Ltd.*) as described previously. Experiments were carried out in triplicate in a parallel manner for each concentration of target compounds and the results were presented as mean ± SE. Control cells were given only culture media. After incubation for 48 h, absorbance (A) was measured at 570 nm. Survival ratio (%) was calculated using the following equation: survival ratio (%)  =  (A_treatment_/A_control_) × 100%. IC_50_ was taken as the concentration that caused 50% inhibition of cell viabilities and calculated by the SAS statistical software.

### Cell morphological assessment

To detect morphological evidence of apoptosis, HCT116, MCF-7 and SKBR3 cell nuclei were visualized following DNA staining with the fluorescent dye DAPI. Briefly, cells were seeded at a concentration of 1×10^5^ cells/well in 6-well tissue culture plates and treated with indicated concentration of **S13**. At the end of incubation, the morphology of cells was monitored under an inverted light microscope. Cells were then fixed with 4% paraform for 20 min and washed with PBS, and then incubated with DAPI (1 μg/ml) for 10 min. After washed with PBS, cells were observed using fluorescent microscopy (Olympus, Japan) with a peak excitation wave length of 340 nm.

### The preparation of Hsp90

The region encoding full-length Hsp90 was subcloned into pET28a. Protein expression in E. coli cells were induced with 0.5 mM IPTG. Cells were harvested after 20h of growth at 16°C and then disrupted by sonication. The soluble lysate was clarified by centrifugation and applied to a Ni^2+^-nitrilo-triacetic acid (NTA) agarose column in a buffer (50 mM Tris-Cl, 300 mM NaCl, 10 mM Imidazole, 10% [v/v] Glycerol, 10 mM PMSF, 10 mM DTT). Hsp90 Protein was eluted with a linear gradient of 20–1000 mM imidazole. Hsp90 was identified by SDS-PAGE, and the high concentrated fraction was dialyzed against ATPase buffer (20 mM Tris-Cl, pH 7.5; 6 mM MgCl_2_; 20 mM KCl) and then aliquoted, frozen in liquid nitrogen, stored at −80°C.

The test compounds were diluted from mother plates (10 mM in 100% (v/v) DMSO) into daughter plates (200 μM in 2.0% (v/v) DMSO); 5 μL of test compound solution was added to each well (equivalent to a final concentration of 40 μM) of the 96-well assay plate. The first and last rows of the 96-well plate contained the appropriate concentration of DMSO were used as blank control. ATP was dissolved in the assay buffer to give a stock concentration of 2.5 mM and stored at room temperature. A 10 μL aliquot of ATP solution was added to each well to give a final assay concentration of 1 mM. Before the usage, Hsp90 protein was thawed on ice and suspended in chilled assay buffer to a stock concentration of 0.45 mg/mL, and the solution was kept on ice. The incubation was started by adding 10 μL of the stock Hsp90 to each well (except for the background wells which received 10 μL of assay buffer), giving a final assay volume of 25 μL. The plates were shaken for approximately 2 min and incubated for 3 h at 37°C.

Two methods are used to evaluate the Hsp90 ATPase activity by detecting HSP90 phosphorylation level.

### Malachite Green Assay

The assay procedures were based on the literatures [33]–[35]. The malachite green reagent was prepared and contained malachite green (0.0812%, w/v), polyvinyl alcohol (2.32%, w/v; dissolves with difficulty and requires heating), ammonium molybdate (5.72%, w/v, in 6M HCl), and AR water, mixed in the ratio 2∶1∶1∶2. The reagent is initially dark brown, but on standing for ∼2 h at room temperature changes to a golden yellow and is ready for use. The assay buffer was 100 mM Tris–HCl, 20 mM KCl, 6 mM MgCl_2_, pH 7.4.

To stop the ATPase reactions, 80 μL of the malachite green reagent was added to each well. Following the addition of 10 μl of 34% sodium citrate to each well, the plate was incubated at room temperature for about 15 min, and the absorbance at 620 nm was measured by Varioskan multimode microplate spectrophotometer (Thermo, Waltham, MA, USA).

### ATP Hydrolysis Inhibition

The Discover RX ADP Hunter^TM^ Plus Assay kit (Discoverx, Fremont, CA) was used following the manufacturer's instructions. ATPase reactions were carried out after 3 h at 37°C temperature in presence of different concentrations of compounds. ADP generation was measured using Varioskan multimode microplate spectrophotometer (540 nm excitation and 620 nm emission). Fluorescence intensity values measured for Hsp90 without any testing compound was assumed as 100% of enzyme activity. The background reaction rate was measured in a reaction lacking enzyme or substrate and subtracted from the experimental rates.

### Hsp70 ATPase activity assay

Hsp70 ATPase activity was measured using Discover RX ADP Hunter^TM^ Plus Assay kit assay as descripted in the manufacturer's instructions. Breifly, Hsp70 (3 μM) was incubated with 1 mM ATP in 100 mM Tris pH 7.4 at 37°C for 3 h in the presence or absence of various concentrations of test compounds. ADP generation was measured using Varioskan multimode microplate spectrophotometer (540 nm excitation and 620 nm emission). Fluorescence intensity values measured for Hsp70 without any testing compound was assumed as 100% of enzyme activity. The background reaction rate was measured in a reaction lacking enzyme or substrate and subtracted from the experimental rates.

### Western-Blot analysis

MCF-7 cells were pretreated with various concentrations of the test compounds. After stimulation, cells were collected; lysed in lysis buffer [50 mM Tris-Cl, pH 7.6, 150 Mm NaCl, 1 mM EDTA, 1% (m/v) Nonidet P-40 (NP-40), 0.2 mM Phenylmethanesulfonyl fluoride (PMSF), 0.1 mM NaF and 1.0 mM dithiothreitol (DTT)], and the supernatant was obtained after centrifugation at 13,000× g for 10 min at 4°C. The concentration of protein in the supernatants was measured by the bicinchoninic acid (BCA) assay. Then equal amounts of protein (50 μg) were separated by 8% or 10% sodium dodecyl sulfate polyacrylamide gel electrophoresis (SDS-PAGE) and transferred onto the PVDF membranes (Millipore, Billerica, MA). The blots were incubated with specific antibodies against the indicated primary antibodies overnight at 4°C followed by IRDyeTM800-conjugated secondary antibody for 1 h at 37°C. Detection was performed by the Odyssey Infrared Imaging System (LI-COR; Lincoln, NE). All blots were stripped and incubated with polyclonal anti-β-actin antibody to ascertain equal loading of proteins.

## Result and Discussion

### Pharmacophore modeling

A powerful approach in computer-aided design is the automated generation of pharmacophore models within DS, In this paper, ten hypotheses were generated from the automated analysis of the information of the training set molecules, and Hypo1 is the best significant pharmacophore hypothesis characterized by the highest cost difference, lowest error cost, lowest root mean square divergence with the best correlation coefficient. 10 pharmacophore models displayed approximate pharmacophore features with hydrogen donor, hydrogen acceptors and hydrophobic center (Table 1).

**Table 1:** Results of pharmacophore hypotheses generated by HypoGen.

Hypo^a^	Total cost	Cost diff.^b^	rms deviation	Correlation (r)	Features^c^
Hypo1	79.556	103.179	1.054	0.961	HBA, HBA, HBD, HY
Hypo2	80.008	102.727	1.076	0.960	HBA, HBA, HBD, HY
Hypo3	86.764	95.971	1.289	0.942	HBA, HBA, HBD, HY
Hypo4	96.923	85.812	1.731	0.891	HBA, HBA, HBD, HY
Hypo5	97.842	84.893	1.755	0.888	HBA, HBD, HY, HY
Hypo6	97.856	84.879	1.770	0.885	HBA, HBD, HY, HY
Hypo7	97.994	84.741	1.777	0.884	HBA, HBD, HY, HY
Hypo8	98.479	84.256	1.742	0.890	HBA, HBA, HBD, HY
Hypo9	99.674	83.061	1.830	0.877	HBA, HBD, HY, HY
Hypo10	100.890	81.846	1.843	0.875	HBA, HBD, HY,HY

a Number for the hypothesis are consistent with the numeration as obtained as obtained by the hypothesis generation.

b Cost difference between the null cost and the total cost, the null cost of the 10 top-scored hypotheses is 182.735, the fixed cost value is 69.5474, and the configuration cost is equal to 16.1676.

c Pharmacophore features were encoded by HBA for hydrogen bond acceptor, HBD for hydrogen bond donor and HY for hydrophobic features.

**Figure 3:**
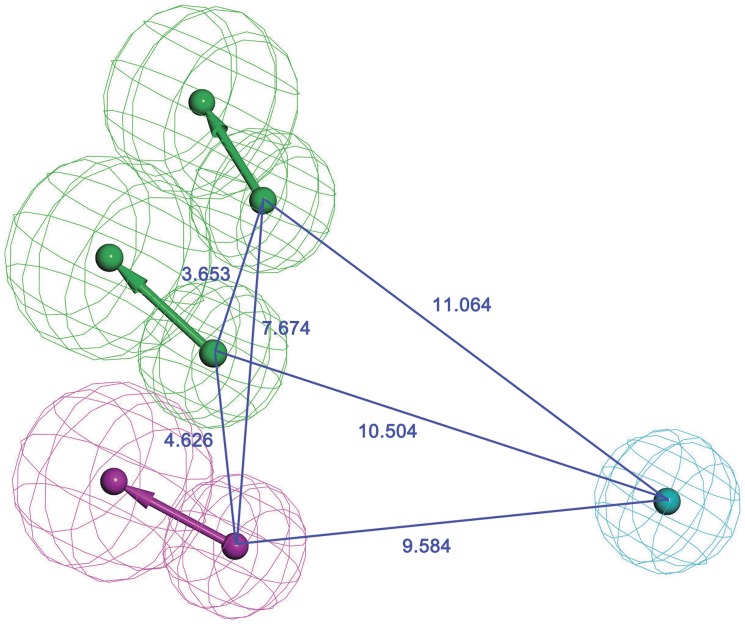
Top scoring pharmacophore model Hypo1 of Hsp90 inhibitors. Hypothesis features are color-coded as follows: hydrophobic aromatic, light blue; hydrogen bond donor, violet; hydrogen bond acceptor, green.

The top-ranked one, Hypo1 (Figure 3), consists of two hydrogen bond acceptors, one hydrogen donor, and one hydrophobic features. In details, the null cost value of the best 10 ranking hypothesis is 182.735, and the fixed cost value is 69.5474. Configuration cost, a constant value less than 17, describing the complexity of the hypotheses space to explore, is 16.1676. As the best model, Hypo1 is characterized by the highest cost difference (79.556), the lowest rms deviation value (1.045) and the best correlation coefficient value (0.961), which represents a true correlation and a good predictability of Hypo1. As a result, it was retained for further analysis.

In addition to the cost analysis, another validation method is to check for Hypo1's capacity to correctly predict the activity of the training set compounds. Except compound **5**, the predicted errors of all the training set compounds were less than 3 (Table 2), indicating that most of the IC_50_ values were predicted correctly.

**Table 2:** The actual IC_50_ value and estimate value predicted by Hypo1 in the training set.

Compd. no.	Fit	IC_50_ (nM)		Errors^a^
		Estimate	Actual	
1	7.68	2.4	6	−2.5
2	6.32	55	29	+1.9
3	6.43	43	37	+1.2
4	6.51	35	39	−1.1
5	5.70	230	40	+5.7
6	6.54	33	56	−1.7
7	5.38	470	200	+2.4
8	5.66	250	230	+1.1
9	5.41	440	280	+1.6
10	4.99	1200	700	+1.7
11	4.39	4700	4700	−1
12	4.59	2900	4800	−1.6
13	4.70	2300	6100	−2.7
14	4.46	3900	6600	−1.7
15	4.59	2900	7100	−2.4
16	3.79	18000	15000	+1.2
17	3.42	43000	20000	+2.2
18	3.97	12000	31000	−2.6

a Value in the error column represents the ratio of the estimated activity to the tested activity or its negative inverse if the ratio is lower the one.

**Figure 4:**
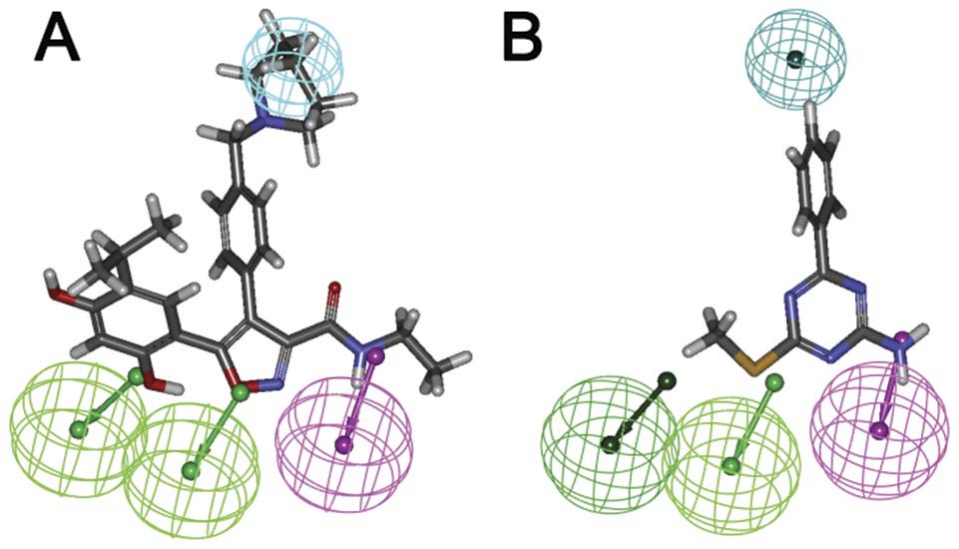
The training set compound 1 (A) and compound 17 (B) mapped onto Hypo1. Hypothesis features are color-coded as figure 3.

Then the most active compound **1** and the very low active compound **17** were mapped onto Hypo1, respectively. Compound **1** fitted all features of Hypo1 very well (Figure 4A). The hydrophobic feature was mapped by the phenyl group, the two hydrogen acceptor features were fitted by the oxygen atom of a phenolic group and imidazole ring, respectively, and the hydrogen donor feature was located on the hydrogen atom of amide. While for the case of compound **17** (Figure 4B), a hydrophobic feature overlapped with the phenyl rings, and the amine served as the hydrogen donor, whereas the two hydrogen features mapping were missing, leading to the dramatically decrease in activity.

**Figure 5:**
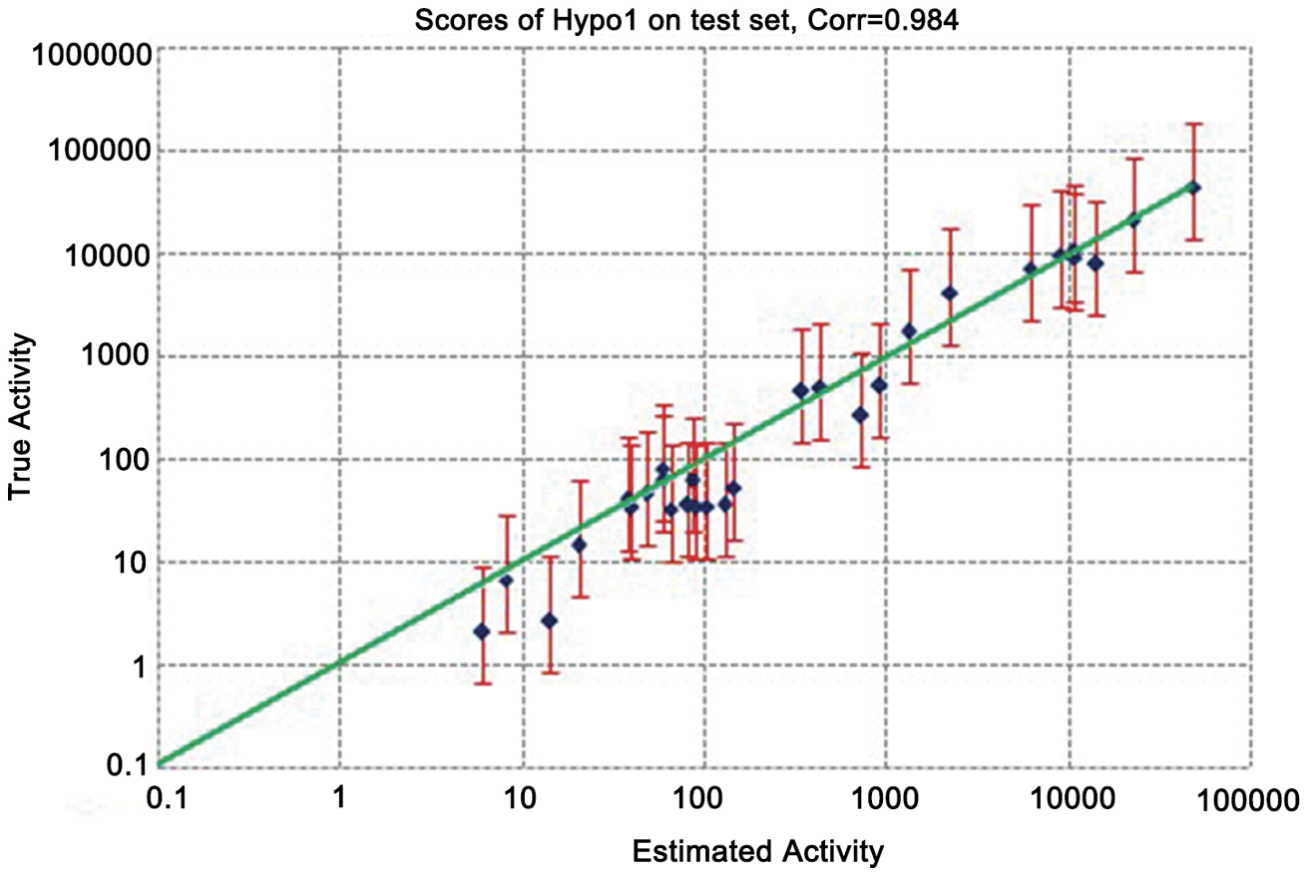
The regression of actual versus predicted activities in the training and test set molecules.

Finally, we applied a test set of 30 molecules with diverse chemical structures (Figure 2) to verify the prediction accuracy of Hypo1. The test set molecules were mapped onto Hypo1 and the actual activity versus estimated activity were calculated (Table 3), and most of members of test set were correctly predicted. We also used Hypo1 to perform a regression analysis with the test set compounds in order to check the predictive ability of this model. Linear regression of the predicted activities versus the experimental ones gave a fairly good correlation coefficient of 0.984 (Figure 5), confirming the prediction accuracy of our model.

**Table 3:** Actual and estimated activities in the test set.

Compd. no.	Fit	IC_50_ (nM)				Errors
		Estimate	Activity^a^ scale	Actual	Activity scale	
19	7.74	2.1	+++	6	+++	−2.9
20	7.623	2.7	+++	14	+++	−5.2
21	7.222	6.8	+++	8	+++	−1.2
22	6.893	14.7	+++	20	+++	−1.4
23	6.543	32.9	+++	64	+++	−2.0
24	6.539	33.2	+++	87	+++	−2.6
25	6.535	33.5	+++	39	+++	−1.2
26	6.517	34.8	+++	102	++	−2.9
27	6.509	35.6	+++	127	++	−3.6
28	6.503	36	+++	80	+++	−2.2
29	6.459	40	+++	37	+++	1.1
30	6.403	45.4	+++	47	+++	−1.0
31	6.331	53.6	+++	142	++	−2.6
32	6.272	61.3	+++	84	+++	−1.3
33	6.263	62.7	+++	58	+++	1
34	6.151	81.1	+++	58	+++	1.4
35	5.637	264.8	++	720	++	−2.7
36	5.407	449.7	++	340	++	1.3
37	5.37	489.6	++	431	++	1.1
38	5.355	507.3	++	914	++	−1.8
39	4.824	1720.5	++	1300	++	1.3
40	4.444	4132.1	++	2200	++	1.9
41	4.207	7122.1	++	6100	++	1.2
42	4.171	7741	++	13900	++	−1.8
43	4.097	9178.5	++	10700	++	−1.2
44	4.073	9700.9	++	9100	++	1.1
45	4.025	10848.9	+	10500	+	1.0
46	3.749	20456.8	+	22300	+	−1.1
47	3.417	43945.6	+	47600	+	−1.1
48	3.324	54632.7	+	56500	+	−1.1

a activity scale: high active (<100 nM, +++); moderately avtive (100–10000 nM, ++) and inactive (>100000 nM, +).

### Evaluating the fit of the pharmacophore to the binding site of the crystal complex of Hsp90 with ligand

**Figure 6:**
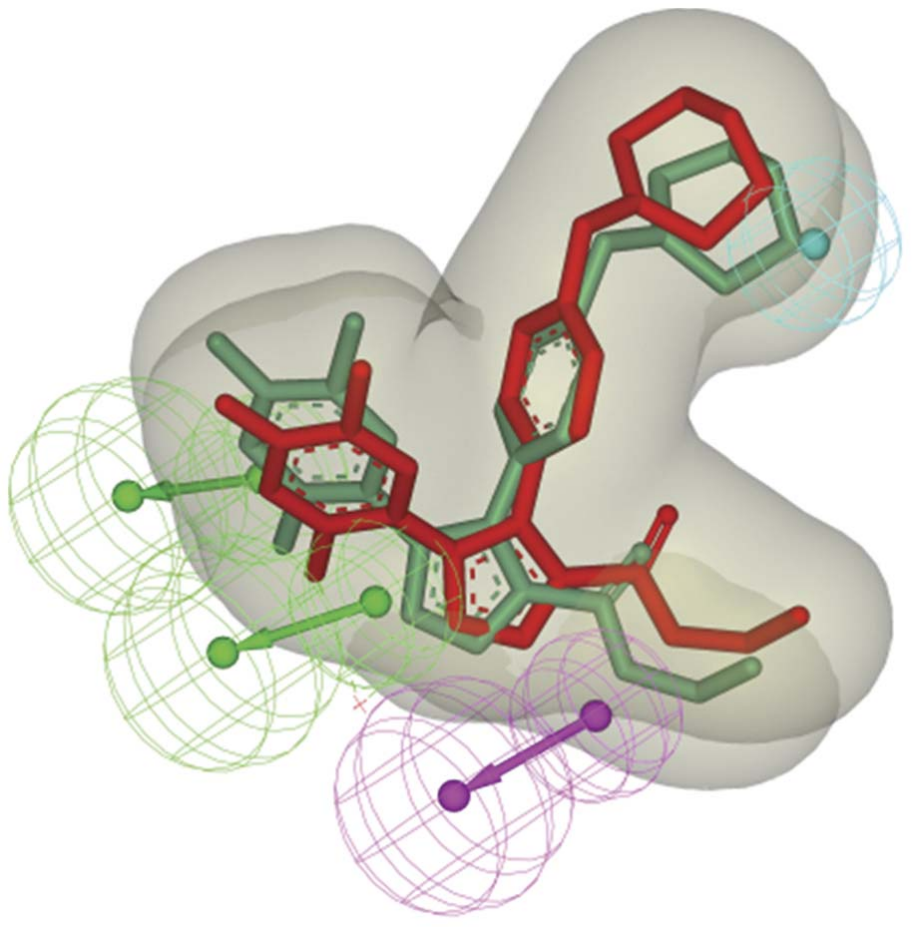
The congruent map of the ligand 2EQ with different conformation. The actual conformation of the ligand 2EQ in the Hsp90 protein crystal complex was shown as red sticks and the active conformation predicted by Hypo1 was shown as green sticks.

We also attempted to evaluate the correlation between of the Hypo1 predicted conformation and the originally bound conformation of a potent Hsp90 ligand. The conformation of the ligand 2EQ (PDB ID: 2vcj) that fitted (Map Ligands to Pharmacophore protocol, ‘best fit’ option) to Hypo1 was compared to the originally bound conformation (Figure 6). The two conformations shared a very similar manner, with the root mean square distance (RMSD) of the heavy atom 0.93 Å. The data indicates the prediction accuracy of Hypo1 in generating the active conformation of potent compounds.

**Figure 7:**
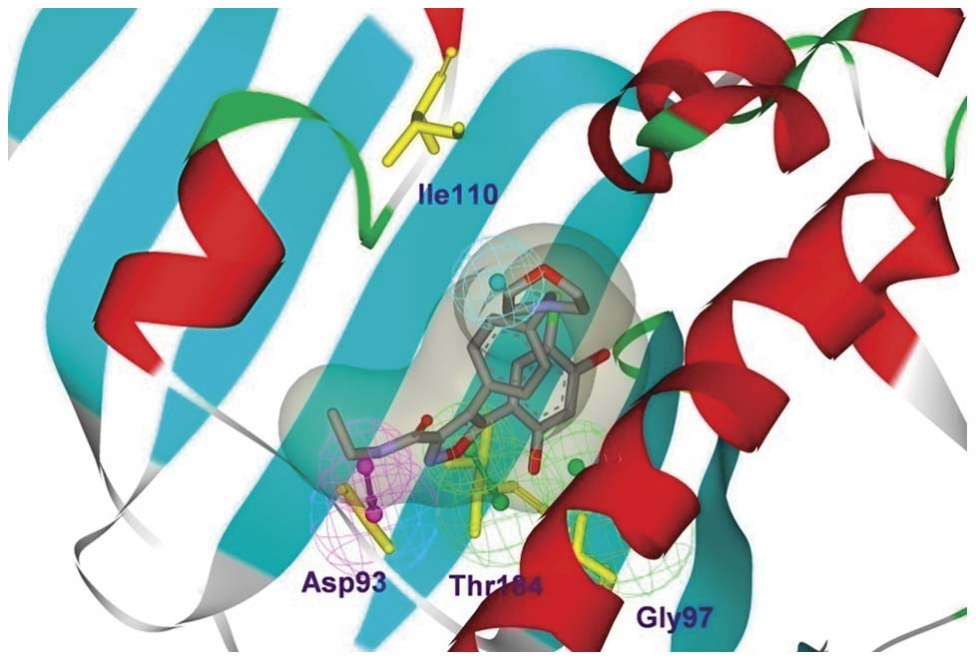
The pharmacophore Hypo1 mapped into the binding site of Hsp90 protein. Ligand and the key residues were shown as stick form and the hypothesis features were color-coded as figure 3.

Additionally, we implemented the pharmacophore model into the active site of Hsp90 crystal structure (PDB id: 2vcj, Figure 7). The pharmacophore Hypo1 is in good agreement with the target-based pharmacophore. Hypo1 appears to accommodate into a narrow tubular pocket of the active site. In the Hsp90 protein crystal structure, the hydrophobic feature of the Hypo1 was located in a hydrophobic cavity formed by residues Gly108, Thr109 and Ile110. The hydrogen bond donor feature pointed to Asp93 and the two acceptor features point to Gly97 and Thr184. Overall, these observations confirm that the proposed ligand-based pharmacophore model can fit into the binding pocket and matches well with the topology of the active site.

### Database screening for potential Hsp90 inhibitors

**Figure 8:**
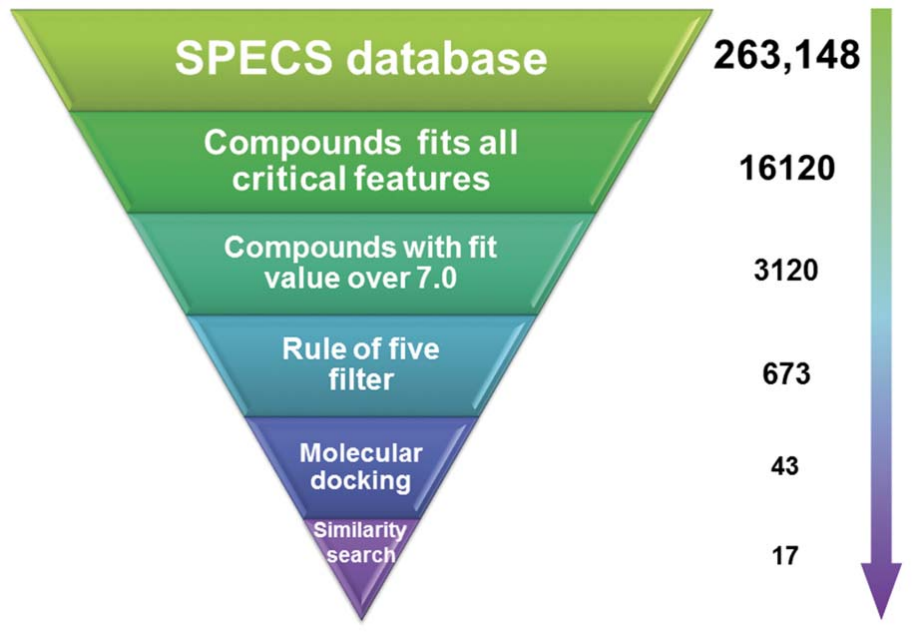
The virtual screening protocol for the identification of novel Hsp90 inhibitors.

To identify novel Hsp90 inhibitors, we built a screening protocol (Figure 8), containing a series of filters, to carry out the virtual screening using Hypo1. SPECS database containing 263,148 compounds was searched using the ‘3D database searching’ protocol in DS. 16120 compounds mapped all critical features in Hypo1 were found and 3210 of them with fit value over 7.0 were retained further analysis. Then an additional filter based on the Lipinski's rule of five was applied for selecting the druggable compounds. After the filtering, we got 673 compounds for further docking studies.

**Figure 9:**
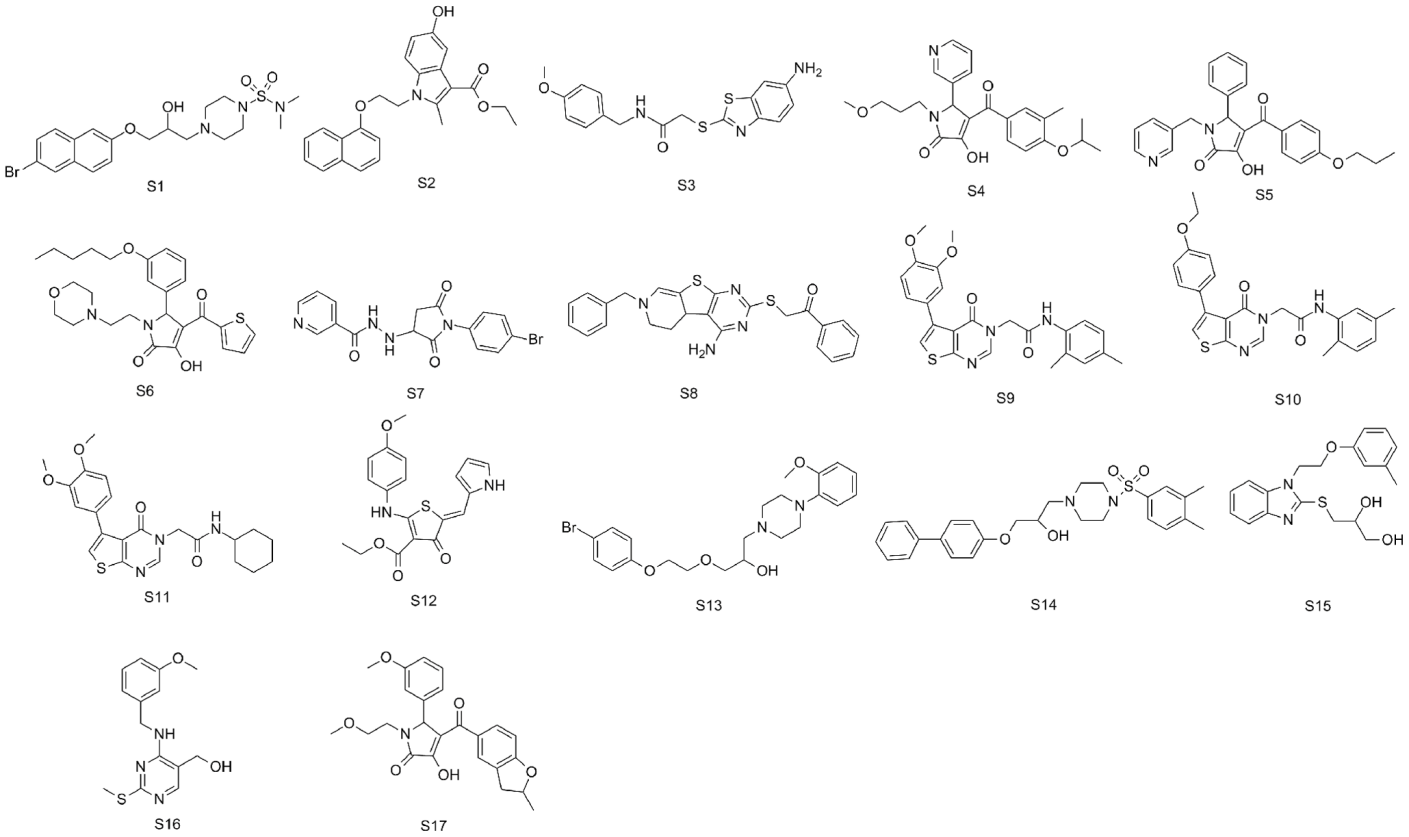
2D Chemical structures of the 17 selected molecules from virtual screening.

According to the experimental section, the crystal structure 3d0b was used to perform the docking study using Gold 5.0. Totally, the top-score 43 compounds with reasonable binding conformations were retained and analyzed carefully to avoid similar structures. Finally, 17 compounds were selected and purchased from SPECS for biological test (Figure 9).

### Biological assay of the hit compounds

To characterize the inhibition of Hsp90 by the 17 compounds, a colorimetric assay for inorganic phosphate was used to measure the ATPase activity of Hsp90. Several compounds (**S4**, **S8**, **S11**, **S14**, **S17**) exhibited moderate Hsp90 inhibition activity. The most potent compounds, **S1** and **S13**, whose inhibition rate were over 50%, were further tested for their IC_50_ (Table 4). The data showed that the two compounds exhibited a dose-dependent manner, with IC_50_ 2.44±0.33 μM and 3.89±0.54 μM. 17-DMAG and **AT13387** were used as positive controls, with IC_50_ 0.95±0.11 μM and 0.37±0.05 μM, respectively.

**Table 4:** Inhibition of Hsp90 of selected 17 compounds.

Compd.	SPECS ID	Inhibition rate (% in 40 μM)	Hsp90 IC_50_ (μM)	Fit Value	Docking Score	
S1	AP-124/43237941	69.71	2.44±0.33^a^ 1.61±0.28^b^	7.97	37.08	
S2	AG-205/37107115	−10.22	ND	7.05	19.94	
S3	AN-465/41674426	−11.55	ND	7.15	24.67	
S4	AF-399/41899907	48.22	ND	7.63	34.24	
S5	AF-399/42099910	−14.46	ND	7.08	15.84	
S6	AF-399/42047618	−7.53	ND	7.11	14.51	
S7	AG-690/13779041	−35.07	ND	7.01	9.48	
S8	AJ-292/11386001	47.77	ND	7.07	38.89	
S9	AP-906/42087482	−47.83	ND	7.03	17.16	
S10	AP-906/42087889	36.04	ND	7.55	38.78	
S11	AP-906/42087549	42.79	ND	7.22	37.05	
S12	AH-487/41949127	12.57	ND	7.17	32.33	
S13	AF-399/42611344	70.58	3.89±0.54^a^ 2.83±0.67^b^	8.06	35.98	
S14	AP-124/43383126	47.53	ND	7.79	34.89	
S15	AG-670/36820009	18.88	ND	7.04	24.61	
S16	AO-638/40907376	−6.22	ND	7.10	18.71	
S17	AA-504/21163113	48.78	ND	7.86	33.50	
17-DMAG		90%	ND	ND	ND	

a by malachite green assay.

b by ADP fluorescence assay.

**Figure 10:**
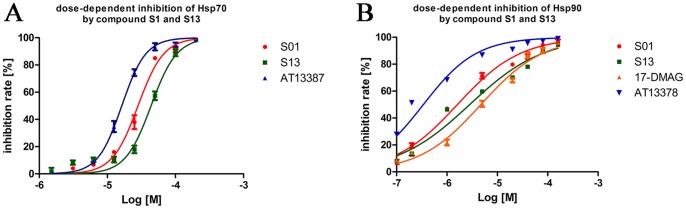
IC_50_ determinations of compound S1 and S13 on heat shock poteins ATPase activity using ADP fluorescence assay. (A) inhibition of HSP70 (B) inhibition of HSP90 The test compounds were diluted from mother plates (10 mM in 100% (v/v) DMSO) into series of concentration (in 2.0% (v/v) DMSO). **AT13387** and **17-DMAG** were used as positive controls in each assay. Data were performed in triplicate and analyzed by GraphPad.Prism.

To confirm the results of malachite green assay, we performed another assay using different method. The Discover RX ADP Hunter^TM^ Plus Assay kit was used for the bioevaluation. The data showed that **S1** and **S13** inhibited Hsp90 with IC_50_ 1.61±0.28 μM and 2.83±0.67 μM. The results confirmed the inhibitory effects of our compounds on Hsp90 (Figure 10).

To evaluate the inhibitory selectivity of our compounds on different kinds of ATPase, we next tested the activities of **S1** and **S13** on the non-GHKL ATPase Hsp70 based on the Discover RX ADP Hunter^TM^ Plus Assay kit. **S1** showed inhibitory IC_50_ 28.20±2.27 μM and **S13** shows inhibitory IC_50_ 43.83±4.38 μM on HSP70 (Figure 10). Both the two compounds inhibit HSP90 over 15 times more potent than HSP70, indicating a target selectivity character of the compounds. Although the selectivity is lower than the positive control **AT13387** (IC_50_ 16.30±1.20 μM and 0.34±0.08 μM on HSP70 and Hsp90, respectively, over 47 times more selective than Hsp70), the two compounds show similar manner with **AT13387**.

Compared to Hsp90, the ATP binding site of Hsp70 has a more polar nature, which requires polar chemical groups in the inhibitor, such as carboxyl group or quaternary ammonium, for the inter-molecular recognition [36]. However, for our compounds, there are no these groups, making the molecules unsuitable to bind with HSP70. This chemical nature of our compounds can also explain the selectivity of our compounds.

**Figure 11:**
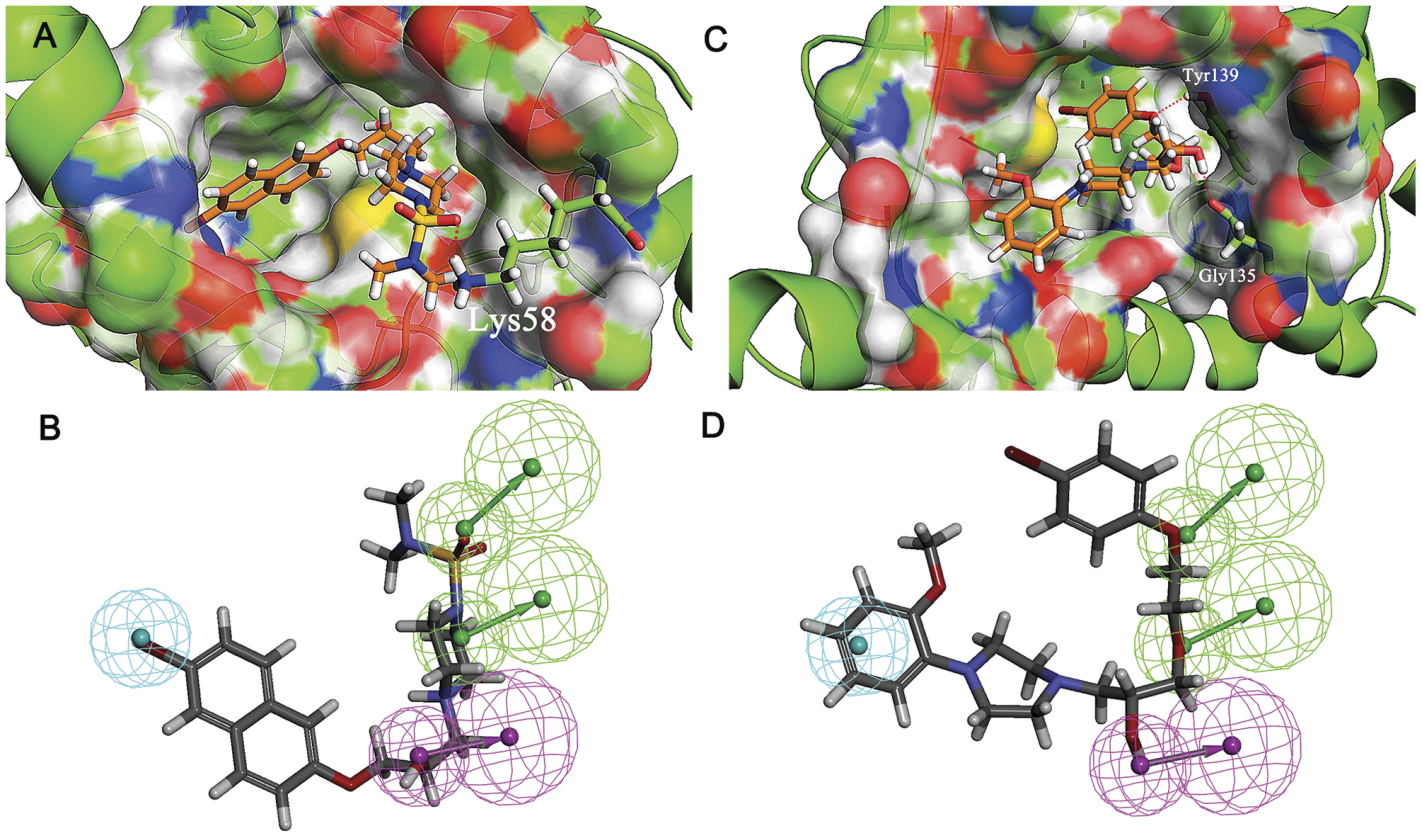
The binding mode analysis of compound S1 and S13 with Hsp90 and pharmacophore model Hypo1. A and C, S1 and S13 binding to the active site of Hsp90, respectively. B and D, S1 and S13 mapped onto the pharmacophore model Hypo1, respectively. S1, S13 and the key residues were shown as stick. H-bond was colored in red. Hypothesis features are color-coded as follows: hydrophobic aromatic, light blue; hydrogen bond donor, violet; hydrogen bond acceptor, green.

The binding modes of **S1** and **S13** to the active site of Hsp90 as well as the Hypo1 were carefully analyzed. For **S1**, naphthalene ring were surrounded by hydrophobic residues Phe138, Tyr139, Val150 and Trp152. the oxygen atom in sulfonamide formed H-bond with Lys58 (Figure 11A). While mapped onto Hypo1, the piperazine ring and the oxygen atom in sulfonamide served as H-bond acceptor, the hydroxyl group was recognized as H-bond donor, and the bromine atom was located on the hydrophobic center (Figure 11B). For **S13**, the hydroxyl group served as H-bond donor, the phenoxy and ethoxy acted as the H-bond acceptor, while the 2-methoxyphenyl ring occupies the hydrophobic center (Figure 11D). While binding to Hsp90 (Figure 11C), the whole scaffold of the molecule inserted well into the binding pocket. The hydroxyl group formed H-bond with Gly135, and the oxygen atom in phenoxy formed another H-bond with Tyr139. The binding modes analysis indicates that **S1** and **S13** can fulfill the requirement of Hsp90 inhibitor in not only structure-based manner but also ligand-based manner, this explains the activities of the compounds.

**S1** and **S13** were further tested for their cytotoxicity against a series of cancer cell lines including SKBR3, A549, HCT116, HepG2, MCF-7 and MDA-MB-231 (Table 5). The results showed that the two compounds, especially **S13**, showed potent and dose-dependent proliferation inhibition on SKBR3, MCF-7, A549 and HCT116 cells with high Hsp90 expression level.

**Table 5:** Anticancer activity data of **S1** and **S13** (IC_50_ values in μM).

Compd.	SK-BR-3	A549	HCT116	HepG2	MDA- MB-231	MCF-7
S1	15.75	25.88	4.66	2.27	2.27	3.79
S13	3.32	0.83	3.63	96.56	14.64	2.01
17-DMAG	1.21	0.057	0.26	0.49	0.048	0.037

**Figure 12:**
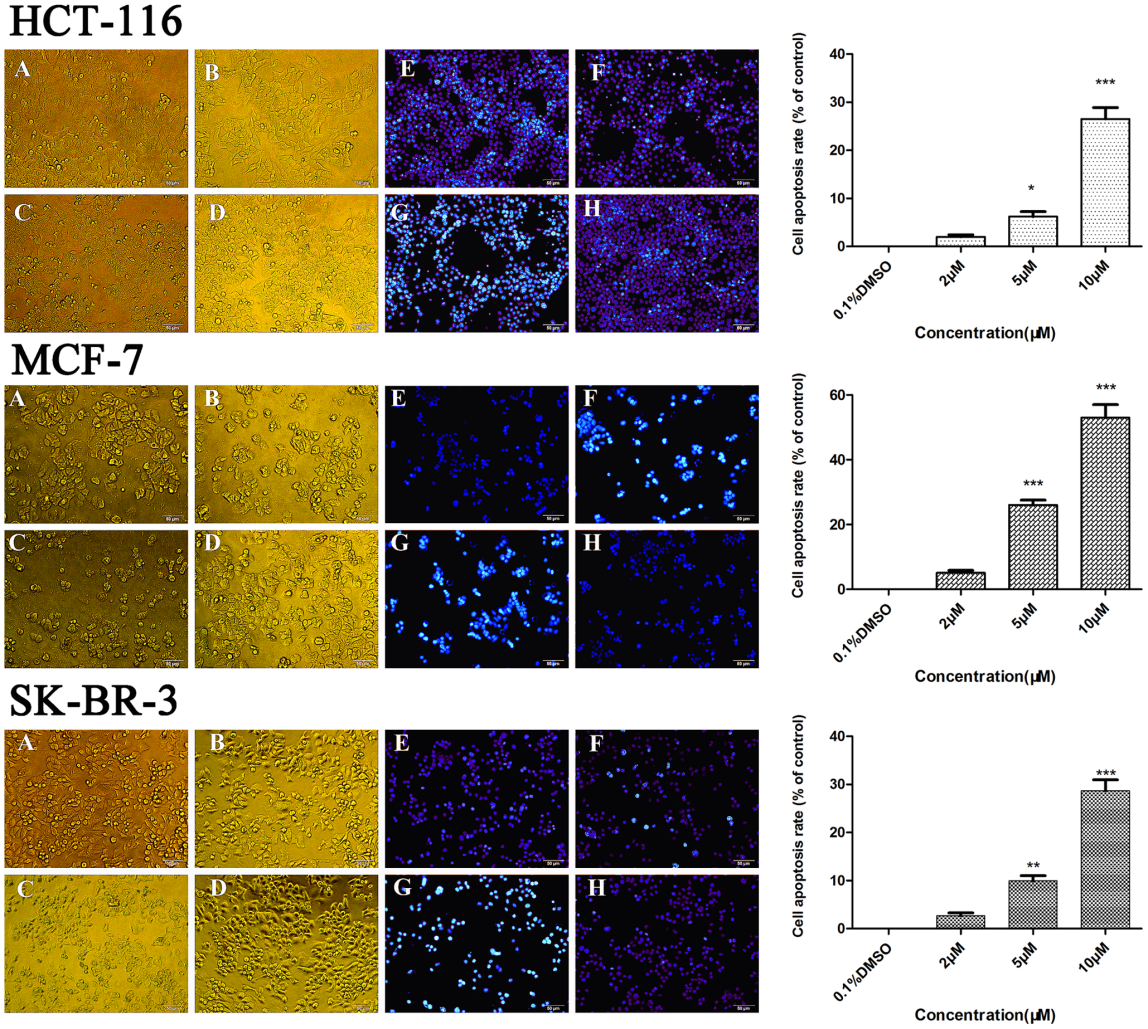
Morphologic changes of the whole cancer cells (A–D) and the nuclei (E–H) induced by compound S13. Cultured HCT116, MCF-7 and SkBR3 cells were treated with compound **S13** at the indicated concentrations. The cells were then were stained with DAPI and observed by fluorescence microscopy, as described in Experimental section. A, 2.0 μM of **S13**; B, 5.0 μM of **S13**; C, 10.0 μM of **S13**; D, Control group; E, 2.0 μM of **S13**; F, 5.0 μM of **S13**; G, 10.0 μM of **S13**; H, Control group.

Compound **S13** was then evaluated for its influence on cell skeleton by a morphological observation study. Under the inverted light microscope (×200), incubation of 2 μM, 5 μM and 10 μM of **S13** for 24 h resulted in phenotypic changes of HCT116, MCF-7 and SK-BR3 cells, such as distortion, membrane blebbing and shrinkage, and a large proportion of cells became round in shape and necrosis at high concentrations, while cells in untreated group grew well and their cytoskeletons were clear (Figure 12). The fluorescence microscopic analysis (×200) presented significant morphological changes of early apoptosis when treated with **S13**. Being identified by DAPI staining, the bright nuclear condensation and the apoptotic bodies appeared after treatment with **S13**, while the untreated cells displayed normal shape and clear skeleton (Figure 12). From the quantification we can observe the dose-dependent apoptosis-induced effects of **S13** in all the tested cell lines, and over 50% of apoptosis is induced by 10 μM **S13** in MCF-7 cells (Figure 12). The results confirm the inhibitory effect of our discovered compounds against Hsp90 on a cell-based level, indicating them as promising leads for novel anti-cancer agents.

**Figure 13:**
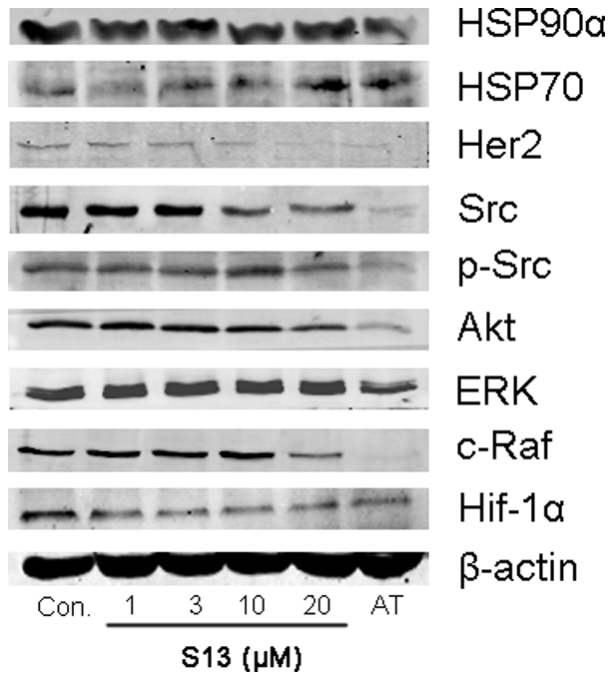
Western-Blot analysis of the expression level of Hsp90, Hsp70 and several client proteins of Hsp90 after incubation with S13 in MCF-7 cells. S13 was incubated with MCF-7 cancer cells at concentrations (μM) for 36 h indicated in the figure **S13** was evaluated for its ability to downregulate the client protein as described in the Experimental Section. Cell extracts were prepared and equivalent amounts of protein were separated by SDS-PAGE and subsequently western-blotted for the indicated proteins.

To further characterize **S13** as a potential Hsp90 inhibitor, MCF-7 cells were treated with varying concentrations of **S13** for 36 h, and equivalent amounts of protein from cell extracts were Western blotted for Hsp90, Hsp70 and a series of client proteins of Hsp90, including Her2, Src, Akt, ERK, c-Raf and Hif-1α, using β-actin as a loading control, and DMSO as a negative control. **S13** was observed to deplete MCF-7 cells of the Hsp90-dependent client proteins in a concentration-dependent fashion (Figure 13), which was in a similar manner with the IC_50_ value for inhibition of the proliferation of the cell line induced by **S13**. Meanwhile, **S13** dose-dependently up-regulates Hsp70. These data all confirm that S13 inhibits the activity of Hsp90, leading to the misfolding of the client proteins, which finally degraded by ubiquitin-proteasome pathway. The results further support the enzyme-based and cell-based evaluation data and indicate that the anti-proliferative effect of **S13** on cancer cell growth is mediated, at least in part, by its ability to inhibit Hsp90.

### Design of new derivatives based on lead compound S13

In order to obtain more potent compounds with improved druggability, compound **S13** was chosen as lead for further molecular modification.

**Figure 14:**
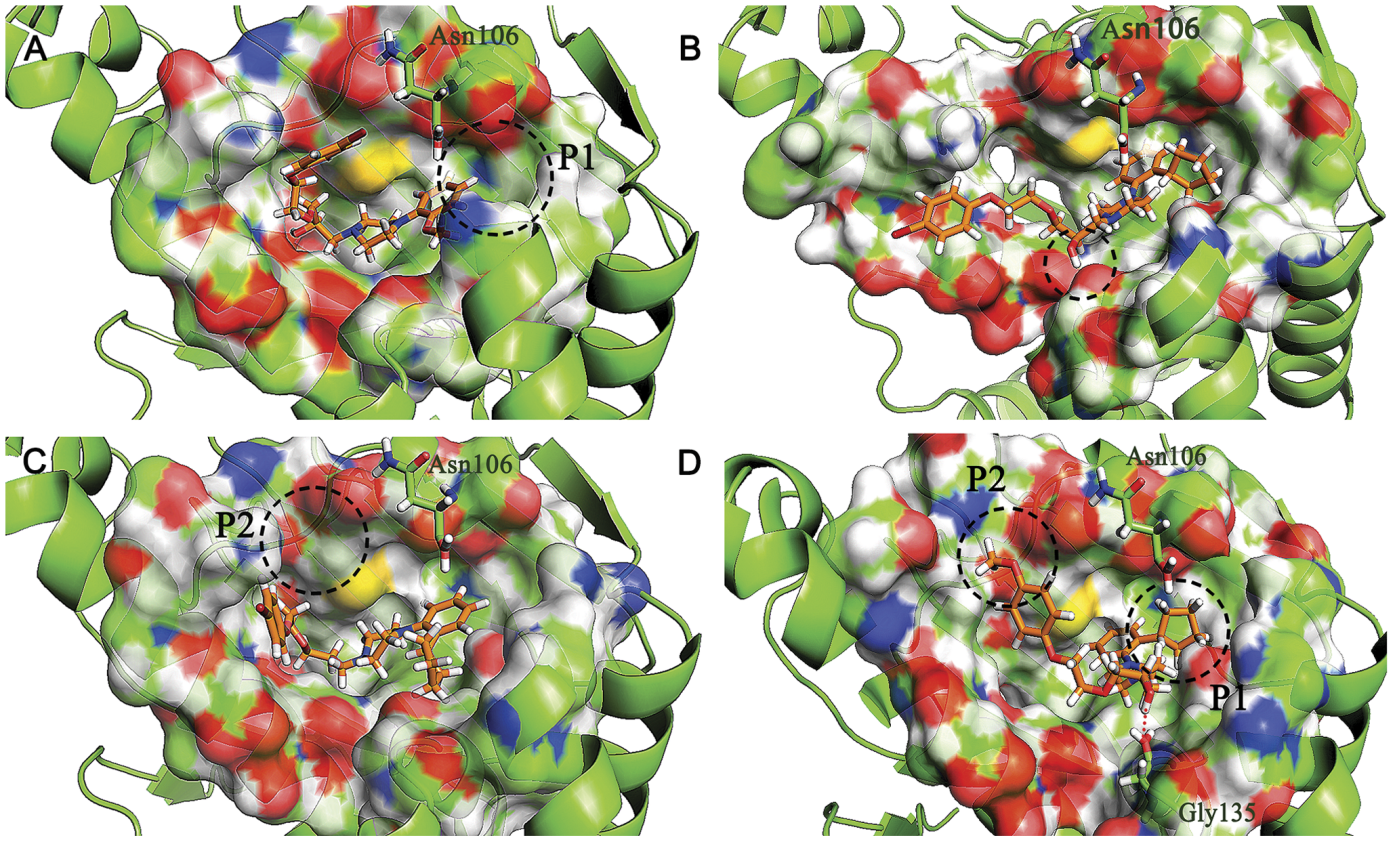
The binding patterns of compound S13 (A), S27 (B), S31 (C) and S40 (D) in the active site of Hsp90.

**Figure 15:**
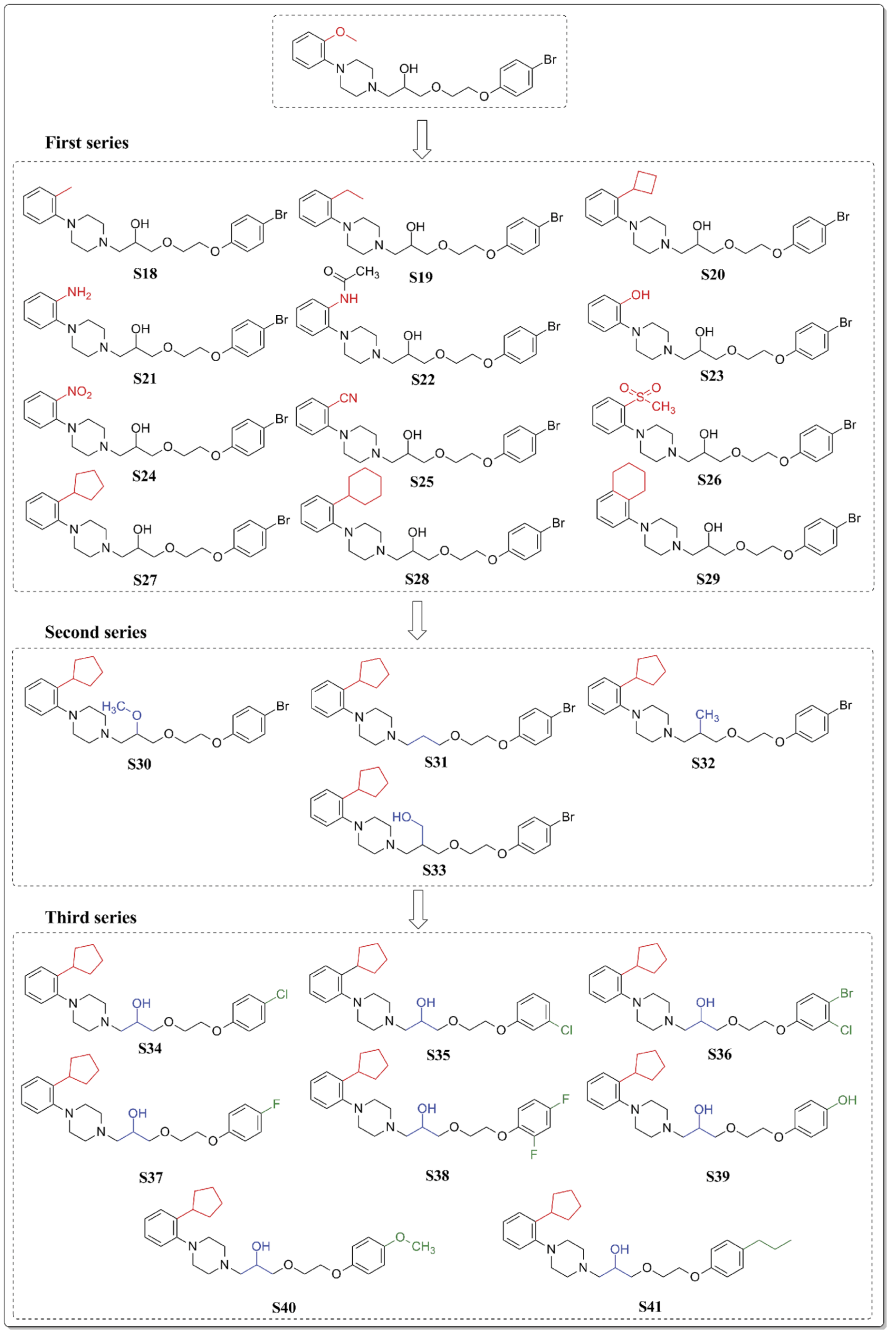
The molecular design strategy and the 2D chemical structures of the designed compounds S18–S41.

Although **S13** bind well to Hsp90, it only occupied part of the binding site, missing the occupation of the hydrophobic sub-pocket P1 (Figure 14A). To solve this problem, we tried to change the methoxy on the phenyl ring with different kinds of substituents, including steric, electron-withdrawing and electron-donating group, leading to the first series of compounds (ranging from **S18–S29**, first series in Figure 15). The docking scores of the compounds indicated that the steric groups obviously enhanced the binding abilities (**S20**, **S27–S29**), while neither electron-withdrawing nor electron-donating groups affected the docking score. The enhanced dock score can be explained by the extra occupation of the P1 sub-pocket (Figure 14B). Although **S28** showed the best score, it exhibited too high CLogP (Table 6), this reduced its druggability and might hinder the further development of the compound. As a result, **S27** with the second best score in the first series of compounds were selected as lead for the next modification.

**Table 6:** The physicochemical properties and toxicity prediction of the designed compounds.

Compd. ID	Chemscore	CLogP	PSA	Toxicity^a^
S13	35.98	3.51	54.40	hERG inhibition
S18	37.25	4.18	45.17	hERG inhibition
S19	39.09	4.62	45.17	hERG inhibition
S20	45.09	4.89	45.17	hERG inhibition
S21	39.45	2.83	71.19	hERG inhibition, Methaemoglobinaemia and Skin sensitisation
S22	39.39	2.90	74.27	hERG inhibition and Methaemoglobinaemia
S23	39.58	3.36	65.40	hERG inhibition
S24	36.79	3.60	90.99	hERG inhibition and Carcinogenicity
S25	38.53	3.52	68.96	hERG inhibition
S26	37.12	2.50	79.31	hERG inhibition
S27	48.30	5.33	45.17	hERG inhibition
S28	48.47	5.78	45.17	hERG inhibition
S29	42.99	5.12	45.17	hERG inhibition
S30	44.82	5.98	83.10	hERG inhibition
S31	46.94	6.02	24.94	hERG inhibition
S32	47.87	6.50	62.87	hERG inhibition
S33	42.28	5.33	45.17	hERG inhibition
S34	44.99	5.17	45.17	hERG inhibition
S35	45.32	5.17	45.17	hERG inhibition
S36	46.81	5.94	45.17	Carcinogenicity and hERG inhibition
S37	46.30	4.71	45.17	hERG inhibition
S38	45.04	4.85	45.17	hERG inhibition
S39	44.00	4.26	65.40	Carcinogenicity, Chromosome damage, Skin sensitisation and hERG inhibition
S40	48.71	4.41	54.40	hERG inhibition
S41	45.46	5.97	45.17	hERG inhibition

a possibility ‘PLAUSIBLE’ in Derek 2.0.3.

We then explored the significance of the hydroxyl group of **S13**. Although it served as the H-bond donor, we thought it may be easily oxidized in vivo, making the molecule unstable in metabolism. Therefore, the second series of compounds with other kinds of groups substituting the hydroxyl were designed (**S30–S33**, second series in Figure 15). The docking score of the four compounds decreased a little compared to **S27**. Meanwhile, the CLogP increased obviously, which may lead to the poor permeability and absorption of the compounds. While binding to Hsp90 (Figure 14B), the hydroxyl oriented to the polar region of the active site, forming H-bond with Gly135, however, this polar contact was missing when compound **S31** bind to Hsp90 (Figure 14C), lowering the binding affinity. All these data proved the significance of the hydroxyl group.

While docking **S27** to Hsp90, we found it missing another polar sub-pocket (P2, Figure 14C) adjacent to the main pocket. With the information in hand, we finally designed the third series of compounds (**S34–S41**, third series in Figure 15), focusing on the substituents on the phenyloxyl group of **S13**. Different halogens, -OH, -OMe and n-propyl were introduced to the phenyloxyl ring. According to the docking results, compound **S40**, substituted by -OMe, exhibited the highest docking score. The binding pattern to Hsp90 showed that the -OMe inserted into the sub-pocket P2 as our expected (Figure 14D). The CLogP and PSA of **S40** was 4.41 and 54.40, respectively, indicating an acceptable solubility and druggability. However, according to the Derek predication, all of the designed compounds had potential hERG inhibition effect. This may due to the similarity of the scaffold of the designed compounds to the pharmacophoric structure of hERG inhibitors. The problem needs further biological validation. In summary, the binding affinity, physicochemical properties make **S40** a proper lead for the design of novel Hsp90 inhibitor.

## Conclusion

By the means of ligand based drug design, a 3D quantitative pharmacophore model with reliable prediction ability was established in the present study. By using the model, two active compounds in both Hsp90-based level and cell-based level were identified from the virtual screening. Further biological evaluation of **S13** revealed that it induced apoptosis while treated with HCT116, MCF-7 and SK-BR-3 cancer cells, which may be through the downregulation of the client proteins of Hsp90. To obtain compounds with improved potency and druggable characters, we designed 24 derivatives of **S13** by a fully consideration of their binding affinities, physicochemical properties and toxicities, resulting a more proper lead compound **S40**. However, during the toxicity prediction we observed the potential hERG inhibitory effects of the series of compounds. The results need to be validated in future by hERG inhibition assay and more careful consideration of the molecular design strategy may help us to avoid the problem. In summary, **S40** can serve as a promising lead for further chemical modification and optimization.
